# Identification of Differently Expressed mRNAs in Atherosclerosis Reveals CDK6 Is Regulated by circHIPK3/miR-637 Axis and Promotes Cell Growth in Human Vascular Smooth Muscle Cells

**DOI:** 10.3389/fgene.2021.596169

**Published:** 2021-02-15

**Authors:** Le Kang, Hao Jia, Ben Huang, Shuyang Lu, Zhenhang Chen, Jinqiang Shen, Yunzeng Zou, Chunsheng Wang, Yongxin Sun

**Affiliations:** ^1^Department of Cardiac Surgery, Zhongshan Hospital, Fudan University, Shanghai, China; ^2^Central Laboratory of Cardiovascular Institute, Zhongshan Hospital, Fudan University, Shanghai, China

**Keywords:** circHIPK3, MiRNA-637, atherosclerosis, CDK6, VSMCs

## Abstract

Atherosclerosis is the leading cause of heart disease and stroke, and one of the leading causes of death and disability worldwide. The phenotypic transformation of vascular smooth muscle cells (VSMCs) plays an important role in the pathological process of atherosclerosis. The present study aimed to identify differently expressed mRNAs in atherosclerosis by analyzing GSE6088 database. Our results revealed there were totally 467 increased and 490 decreased differential expressed genes (DEGs) in atherosclerosis. Bioinformatics analysis demonstrated that the DEGs substantially existed in pathways, including Glyoxylate and dicarboxylate metabolism, Tyrosine metabolism, Tryptophan metabolism, Beta-Alanine metabolism, Fatty acid biosynthesis and Starch and sucrose metabolism. Next, we constructed a protein-protein interaction (PPI) network to identify hub genes in atherosclerosis. Also, we identified CDK6 as a key regulator of atherosclerosis. In this study, we found that CDK6 knockdown suppressed HASMC and HUASMC cell proliferation. Circular RNA (CircRNA) is a non-coding RNA which is reported to have an unusual influence on tumorigenesis process and other aspects in the last few years. Previous studies showed circRNAs could act as miRNAs sponging in multiple biological processes. Bioinformatics prediction and luciferase analysis showed that CDK6 were targeted and regulated by circHIPK3/miR-637. Moreover, silencing circHIPK3 could also significantly induce the arrest and apoptosis of cell cycle. In conclusion, this study discovered the important regulatory role of circHIPK3 in the proliferation and apoptosis of VSMCs by influencing the miR-637/CDK6 axis.

## Background

Atherosclerosis is a very common chronic progressive inflammatory disease which results in many cardiovascular ailments, such as coronary heart disease, cerebral infarction and so on (Lahoz and Mostaza, 2007). Atherosclerosis usually refers to the stenosis or obstruction of the vascular lumen due to the formation of atherosclerotic plaque on the coronary artery wall, and then causes local insufficient blood supply and increased lumen pressure (Pasterkamp et al., 1995; Falk, 2006). Sustained insufficient blood supply and overpressure can lead to multiple organ involvements and failures (Landow and Andersen, 1994; White, 1996). The formation of atheromatous is related to endothelial, vascular smooth muscle cells (VSMCs), foam cell and cytokines (Falk, 2006). Chronic inflammation has a significant role in atherosclerosis (Shishehbor and Bhatt, 2004). However, human endothelial cells and VSMCs can produce these proinflammatory mediators in the progression and development of atherosclerosis. Atherosclerosis occurs after endothelial cell damage by a variety of stimuli including hypertension, dyslipidemia and etc. After the initial injury, different types of cells such as inflammatory cells, platelets and endothelial cells itself release mediators (cytokines and growth factors) causing multiple effects. These mediators will promote the transition of VSMCs from a quiescent contraction state to an active synthetic state, promote the proliferation and migration of VSMCs (Libby et al., 1997; Schachter, 1997). VSMCs exist in different phenotypes under different conditions. Physiologically, the primary VSMCs phenotype is the quiescent contractile state also termed as differentiated VSMCs which performing contractile function to regulate blood vessel diameter (vasoconstriction and vasodilation) and blood flow. Besides, VSMCs can transform from differentiated phenotype to dedifferentiated phenotype in response to various stimuli. The dedifferentiation phenotype is the active synthetic VSCMs which are characterized by increased proliferation/migration ability and decreased contractility (Owens, 1995). During the development of pathogenic vascular remodeling such as arteriosclerosis, VSMCs with dedifferentiation phenotype cause intimal vascular lesions. Therefore, the phenotype transformation of VSMC has an important impact on the formation of atheromatous plaques (Rudijanto, 2007).

Abnormal proliferation of VSMCs plays a key role in the pathogenesis of cardiovascular diseases, such as atherosclerosis and coronary heart disease (Davis-Dusenbery et al., 2011). In order to better understand the etiology of atherosclerosis well, it is necessary to explore the mechanism VSMCs in atherosclerosis. Some cytokines, such as leptin, have been reported to stimulate the proliferation and migration of VSMCs, which is crucial in restenosis (Bodary et al., 2007). Previous studies have shown that uncontrolled cell proliferation may be caused by the imbalance of cell cycle-related proteins such as cyclins and cyclin-dependent kinases (CDKs). CDK6 is a member of the CDKs family, which mainly mediates the regulation of cell cycle progression (Grossel and Hinds, 2006). CDK6 gene has been reported to be overexpressed in a variety of human cancer, such as lymphoid malignancies and gastric cancer (Nebenfuehr et al., 2020).

MicroRNA (miRNA) is a non-coding RNA with a length of approximately 20 nucleotides which are involved in a variety of biological processes, including cell proliferation and apoptosis. Many studies have confirmed that miRNAs play a vital role in the behavior of VSMCs during the process of atherosclerosis. For example, miR-499a-3p and miR-135b-5p can enhance VSMCs proliferation in atherosclerosis through targeting MEF2C (Xu et al., 2015). MiR-126 can alleviate endothelial cell injury through PI3K/Akt/mTOR signaling pathway (Tang and Yang, 2018). MiR-637 has been reported to play a role in a variety of human diseases especially in cancer. For instance, miR-637 suppresses the proliferation and invasion of hepatoma cells by down-regulating AKT1 (Du and Wang, 2019) and inhibits melanoma progression through PTEN/AKT signaling pathway (Zhang et al., 2018). However, few reports have studied the specific role and regulatory mechanisms of miR-637 in atherosclerosis.

Increasing numbers of circular RNAs (circRNAs) have been discovered in eukaryotes because RNA-Seq technology becomes easier and cheaper, with the invention of new sequencing technology (Salzman et al., 2012). CircRNAs are non-coding and closed single-stranded RNA transcripts. However, the circRNAs are once thought to be by-products or a splicing error of the transcriptome because of the limitations of previous sequencing technology (Chen and Yang, 2015). In recent years, researches on circRNAs have become a hotspot due to its specific ring structure. Additional evidence suggests that circRNAs play many important roles in various biological processes. For example, Chen et al. (2017) revealed that CircRNA_100290 exerted regulatory functions in oral squamous cell carcinomas (OSCCs) via control miR-29b/CDK6 axis signaling pathway. Abdelmohsen et al. (2017) revealed that circPABPN1 bound extensively to HuR, which was a common RNA-binding protein, prevented HuR from binding to PABPN1 mRNA and reduced the expression of PABPN1 gene in human cervical carcinoma cells. Besides, some circRNAs play roles as protein scaffolds. Du et al. (2017) found that circFoxo3 promoted MDM2-induced p53 degradation because it had binding sites for MDM2 and p53 in mice. However, knowledge of the role of circRNA in human VSMCs, especially circHIPK3, still needs to be investigated in the future.

This investigation aimed to illustrate the role and mechanism of circHIPK3 in human vascular smooth muscle (HVSMC) cells. We found that circHIPK3 was up-regulation in HVSMC by qRT-PCR. Knockdown of circHIPK3 inhibited the proliferation of VSMCs (HASMC and HUASMC) through CCK-8 assay. We showed that knockdown of circHIPK3 promoted the arrest and apoptosis of cell cycle in VSMCs. These findings imply the possibility of circHIPK3 regulation for growth of VSMCs.

## Materials and Methods

### Microarray Analysis

The gene expression information of GSE6088 was acquired from NCBI GEO^1^. The original data were treated by interactive web tool GEO2R^2^ employing the limma R packages from the Bioconductor project. Differentially expressed genes (DEGs) with *P* < 0.01 were considered as statistically significant and the cutoff criterion.

### Gene ontology and KEGG Pathway Enrichment Analysis

Gene ontology (GO) term and Kyoto Encyclopedia of Genes and Genomes (KEGG) pathway analysis were conducted by an online gene function classification tool called DAVID^3^, which aimed to find the potential genes at the functional level. The cutoff criterion was *P* < 0.05.

### PPI Network and Module Analysis

DEG-encoded proteins and protein-protein interaction (PPI) network were developed using STRING database^4^. Confidence score was considered significant if it was over 0.4. Cytoscape software (Version 3.6.0) (The Cytoscape Consortium, New York, NY, United States) was applied to visualize the PPI network.

### Cell Culture and Transfection

In our experimental system, we employed VSMCs (HASMC, HUASMC) which were obtained from ATCC or preserved in our laboratory, when necessary RPMI-1640 medium (BI, Israel) with 10% fetal bovine serum (Life Technologies, United States) was used to culture the cells in a 37°C incubator with 5% CO_2_.

The siRNA against the spliced junction site of circHIPK3 was collected from Gene-Pharma (China), and lipofectamine 2000 (Invitrogen, United States) was used for transfection according to the manufacturer’s instructions. MiR-637 mimic and inhibitor (GenePharma, China) were used.

### RNA Extraction and qRT-PCR Analysis

Trizol (Takara, Dalian, China) was employed to extract total RNA. We used individual primer directly to flank the head-to-tail splice site. SYBR Green Real-time qPCR was employed for the amplification of cDNA on StepOne Real-Time PCR System (Applied Biosystems) by using SYBR Green PCR kit (TaKaRa). 2^–ΔΔ*CT*^ method was used to analyze the expression of genes.

### Cell Proliferation Assays

Cell Counting Kit-8 (CCK-8) (Beyotime, Shanghai, China) was used to detect cell proliferation. We seeded the transfected cells at a concentration of 1500 cells/well in 96-well plates. At 0, 24, 48, 72, and 96 h respectively, the cell proliferation rates were calculated according to the supplier’s protocol.

### Cell Apoptosis Assays

For the apoptosis assays, VSMCs cells after transfection were added PI and Annexin V under dark conditions and placed on ice for 5min. Flow cytometry on a FACSCalibur Flow Cytometer (BD Biosciences) was conducted to analyze the treated VSMCs cells.

### Luciferase Reporter Assay

Then, wild type and mutant circHIPK3 and CDK6-3′UTR fragments including miR-637 binding sites were reconstituted into the pGL4.10 plasmids (Promega, United States) and subsequently co-transfected by miR-637 mimics or mimics-NC into VSMCs cells with Lipofectamine 2000 transfection reagent. Luciferase activity tests (Promega) were applied according to the manufacturer’s protocols.

### Statistical Analysis

GraphPad Prism 6 was used to analyze the data and all the data were presented as mean ± standard deviation. One-way ANOVA or *t*-test was employed to determine the significant differences between various groups. Log-rank test and Cox regression were performed to analyze Kaplan–Meier curve. *P* < 0.05 was reflected to have statistical significance.

## Results

### Determination of DEGs in Atherosclerosis

**FIGURE 1:**
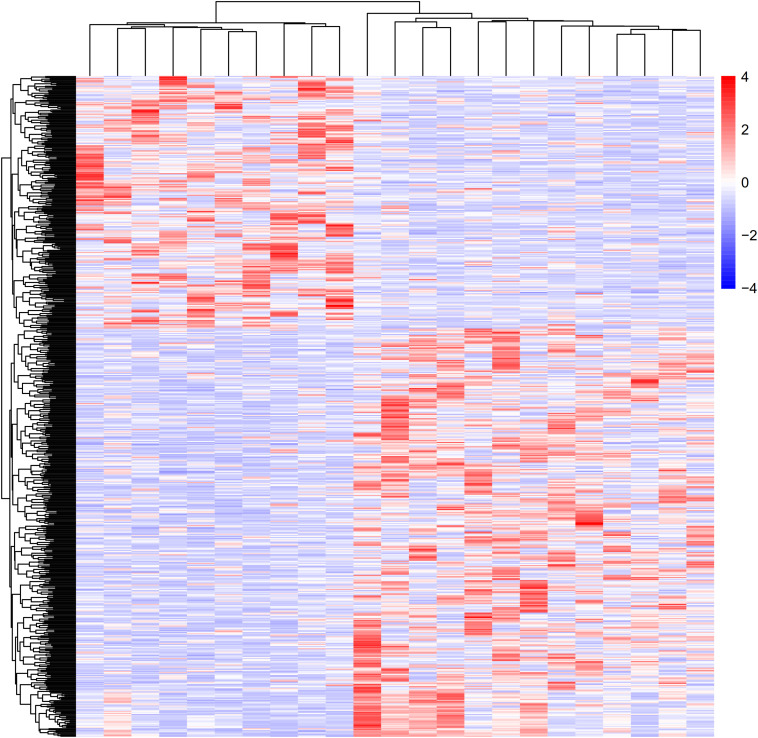
Heat map and cluster dendrogram of DEGs. To determine and analyze the DEGs, NCBI GEO was applied to acquire GSE6088. A total of 3,361 DEGs were at a significant level in atherosclerosis samples compared with normal samples (*P* < 0.01). Red represents increased genes, and blue decreased genes.

To determine and analyze DEGs, NCBI GEO was applied to acquire GSE6088. Based on the *P* < 0.01 cutoff criterion, we filtrated 3361 DEGs in atherosclerosis samples compared with normal samples. A total of 1,672 upregulated DEGs and 1689 downregulated DEGs were obtained from GSE6088 (Figure 1). Top 10 upregulated DEGs were RGS13, PDE1A, RNF17, ARPP21, UTS2, LINC00486, C7orf57, LOC102546226, AL832163, and OSM. Top 10 downregulated DEGs were GJC1, C10orf107, CALB1, KIR2DL1, SCN7A, KIR3DS1, DPY19L2P3, THBS1, FAM221B, and CYP2U1.

### Gene Ontology Analysis and Signaling Pathway Enrichment of DEGs in Atherosclerosis

**FIGURE 2:**
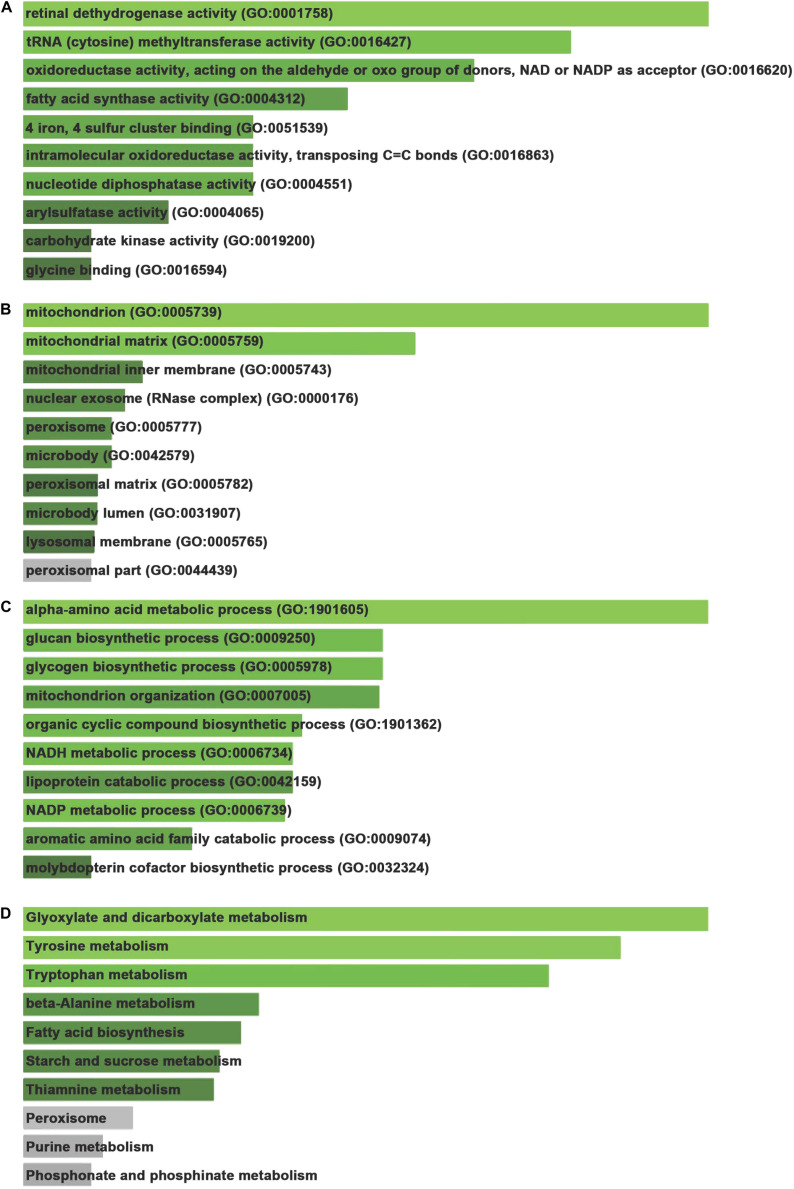
Gene ontology and signaling pathway enrichment of DEGs. **(A)** Molecular function analysis of DEGs. **(B)** Cellular component analysis of DEGs. **(C)** Biological process analysis of DEGs. **(D)** KEGG pathways analysis of DEGs.

To analyze the GO and pathway enrichment, we uploaded all the DEGs to the DAVID online tools. Molecular function analysis showed these DEGs were involved in regulating retinal dehydrogenase activity (GO:0001758), tRNA (cytosine) methyltransferase activity (GO:0016427), oxidoreductase activity, acting on the aldehyde or oxo group of donors, NAD or NADP as acceptor (GO:0016620), fatty acid synthase activity (GO:0004312), 4 iron, 4 sulfur cluster binding (GO:0051539), intramolecular oxidoreductase activity, transposing C=C bonds (GO:0016863), nucleotide diphosphatase activity (GO:0004551), arylsulfatase activity (GO:0004065), carbohydrate kinase activity (GO:0019200), and glycine binding (GO:0016594) (Figure 2A).

Cellular component analysis showed these DEGs were involved in regulating mitochondrion (GO:0005739), mitochondrial matrix (GO:0005759), mitochondrial inner membrane (GO:0005743), nuclear exosome (RNase complex) (GO:0000176), peroxisome (GO:0005777), microbody (GO:0042579), peroxisomal matrix (GO:0005782), microbody lumen (GO:0031907), lysosomal membrane (GO:0005765), and peroxisomal part (GO:0044439) (Figure 2B).

Biological process analysis revealed these DEGs were involved in regulating alpha-amino acid metabolic process (GO:1901605), glucan biosynthetic process (GO:0009250), glycogen biosynthetic process (GO:0005978), mitochondrion organization (GO:0007005), organic cyclic compound biosynthetic process (GO:1901362), NADH metabolic process (GO:0006734), lipoprotein catabolic process (GO:0042159), NADP metabolic process (GO:0006739), aromatic amino acid family catabolic process (GO:0009074), and molybdopterin cofactor biosynthetic process (GO:0032324) (Figure 2C).

Kyoto encyclopedia of genes and genomes pathways analysis demonstrated that DEGs were involved in modulating a series of pathways, including Glyoxylate and dicarboxylate metabolism, Tyrosine metabolism, Tryptophan metabolism, Beta-Alanine metabolism, Fatty acid biosynthesis, Starch and sucrose metabolism (Figure 2D).

### Protein-Protein Interaction Network of DEGs in Atherosclerosis

**FIGURE 3:**
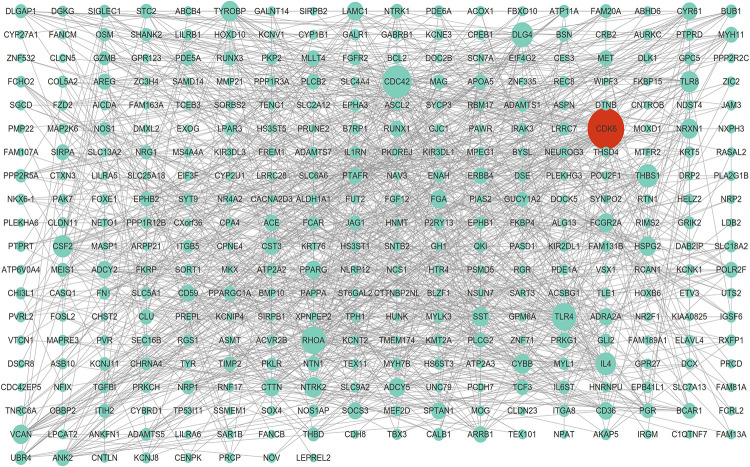
Protein-protein interaction network of DEGs. The highest stringent minimum required interaction score of 0.09 by STRING database was used to construct PPI network including 261 nodes and 1,084 edges.

The STRING database was consulted to anatomize the PPI networks, thus to prove the functional connectivity of the DEGs. We analyzed 2654 DEGs using STRING database, and employed Cytoscape software to build networks on the basis of DEGs. As shown in Figure 3, the PPI network contained 261 nodes and 1084 edges, which demonstrated proteins and interactions.

Among these nodes, we identified a series of key nodes in this network, including CDC42, RHOA, CDK6, TLR4, DLG4, ADCY5, NRXN1, CYBB, TYROBP, and CYR61(Degree top 10). The present study focuses on CDK6, which is a key regulator of cell cycle and plays a crucial role in cell proliferation regulation.

### Suppression of CDK6 Inhibited Cell Proliferation of VSMCs

**FIGURE 4:**
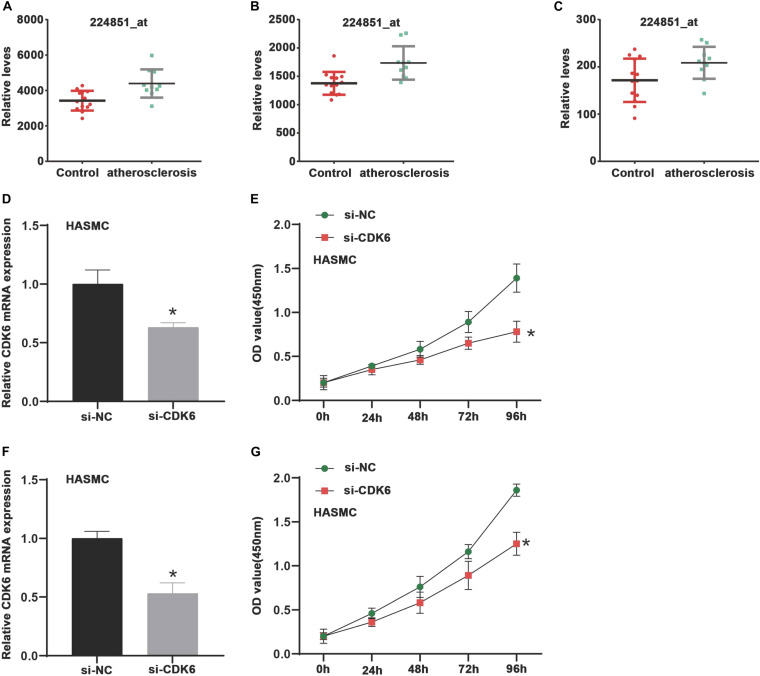
CDK6 is highly expressed in atherosclerosis samples and inhibition of CDK6 reduces cell proliferation *in vitro*. **(A–C)** An online data (GSE6088) was analyzed, and CDK6 expression in atherosclerosis was induced by 1.44-fold compared to normal samples (224851_at, 224847_at, and 243000_at). **(D,F)** RT-PCR analysis of CDK6 mRNA expression levels in HASMC and HUASMC after transfecting with si-CDK6. **(E,G)** Suppression of CDK6 inhibited cell proliferation of VSMCs in HASMC and HUASMC cell. *P < 0.05.

Using the GSE6088 database, the results showed CDK6 expression in atherosclerosis was induced by 1.44-fold compared to normal samples (Figures 4A–C). Aimed at exploring the roles of CDK6 in VSMCs, we detected the impacts of CDK6 knockdown on cell proliferation. HASMC and HUASMC cells were transfected with si-CDK6 and si-NC for 2 days. Then, the levels of CDK6 were detected. As presented in Figures 4D,F, we observed CDK6 was remarkably down-regulated after knockdown of CDK6 in HASMC and HUASMC cells. We used CCK-8 kit to detect the effect of CDK6 on cell proliferation, and the results indicated the cell proliferation rate in CDK6 knockdown cells was reduced compared with normal groups (Figures 4E,G).

### CDK6 Was a Target of miR-637

**FIGURE 5:**
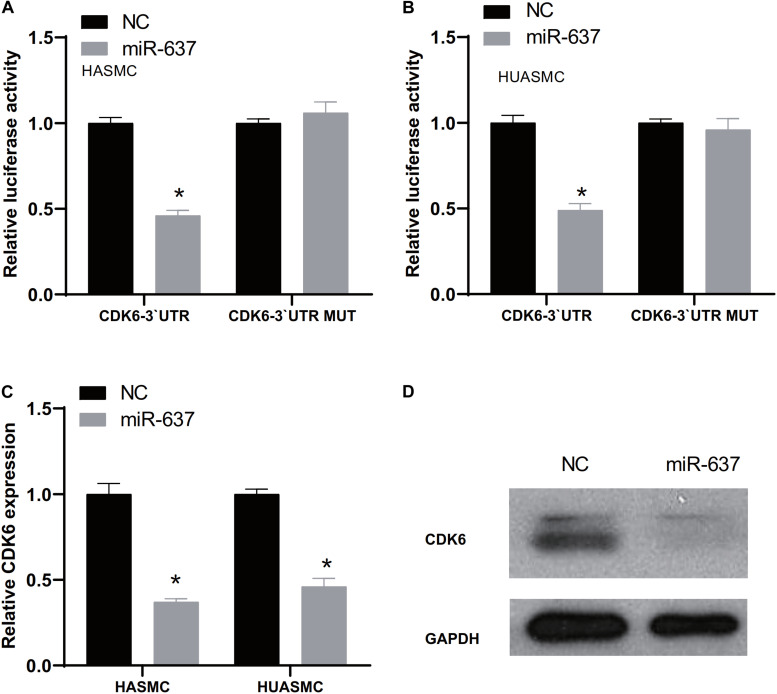
CDK6 was a target of miR-637. **(A,B)** Luciferase assay detects the direct binding of miR-637 on the 3′-UTR of CDK6. **(C)** RT-PCR analysis of CDK6 expression levels in HASMC and HUASMC after overexpression of miR-637. **(D)** Western blot analysis of CDK6 protein levels after overexpression of miR-637. *P < 0.05.

Next, we used TargetScan website^5^ to predict the potential miRNAs regulating CDK6 expression, which was used to explore the upstream regulators of CDK6. The analysis showed a potential target of miR-637 was CDK6. A further luciferase reporter assay demonstrated the direct interaction between miR-637 and CDK6 (Figures 5A,B). The fluorescence signaling in HASMC and HUASMC cells transfected with CDK6 3′-UTR-wt were reduced after overexpression of miR-637. However, the co-transfection of CDK6 3′-UTR-mut and miR-637 did not contribute to the decrease of fluorescence signaling. Then, it was found that both RNA and protein expression of CDK6 were inhibited after overexpression of miR-637 in VSMCs (Figures 5C,D).

### MiR-637/CDK6 Was Regulated by circHIPK3 in VSMCs

**FIGURE 6:**
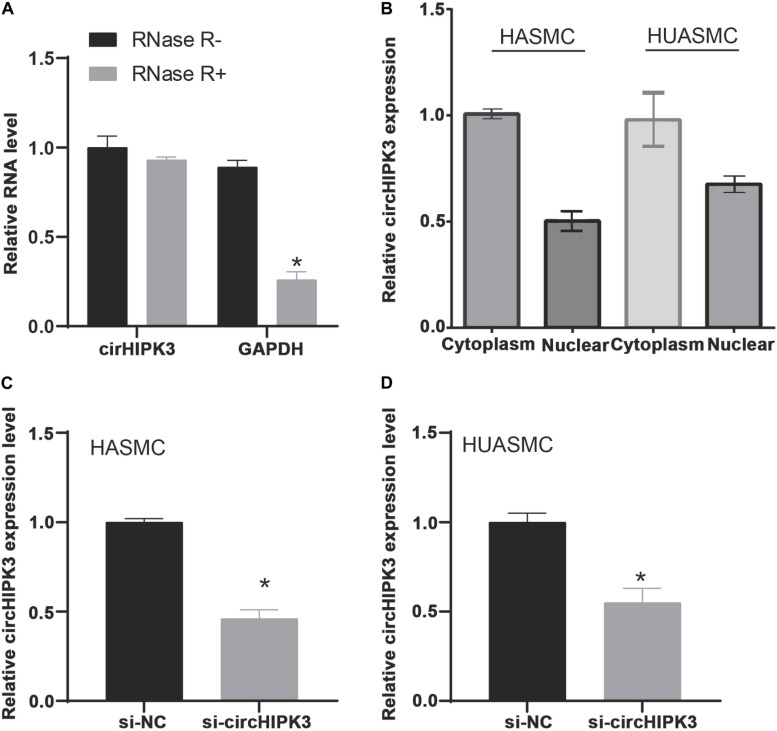
The isolated circHIPK3 is circular structure that is located in the cytoplasm. **(A)** Treated the isolated circHIPK3 with RNase R, circHIPK3 is more resistant to RNase R than linear HIPK3 RNA. **(B)** Subcellular location detection showed that circHIPK3 was mainly located in cytoplasm. **(C,D)** RT-PCR analysis of circHIPK3 expression levels in HASMC and HUASMC after transfecting with si-circHIPK3. *P < 0.05.

Previous studies demonstrated that circRNAs played as miRNA sponges to affect miRNAs’ activities and targets’ expression. Thus, this study applied miRNA target prediction (circBase and StarBase v2.0) to investigate the potential circRNAs that targeting miR-637. We found that circHIPK3 was a potential target of miR-637. As shown in Figure 6A, we used RNase R to treat isolated circHIPK3 to determine the circular structure of this circRNA. The results revealed the circHIPK3 was more tolerant to RNase R treatment than liner HIPK3 RNA. Subcellular location detection showed that circHIPK3 was mainly located in cytoplasm (Figure 6B). Using siRNAs mediated knockdown, we observed circHIPK3 was remarkably down-regulated after knockdown of circHIPK3 in HASMC and HUASMC cells (Figures 6C,D).

**FIGURE 7:**
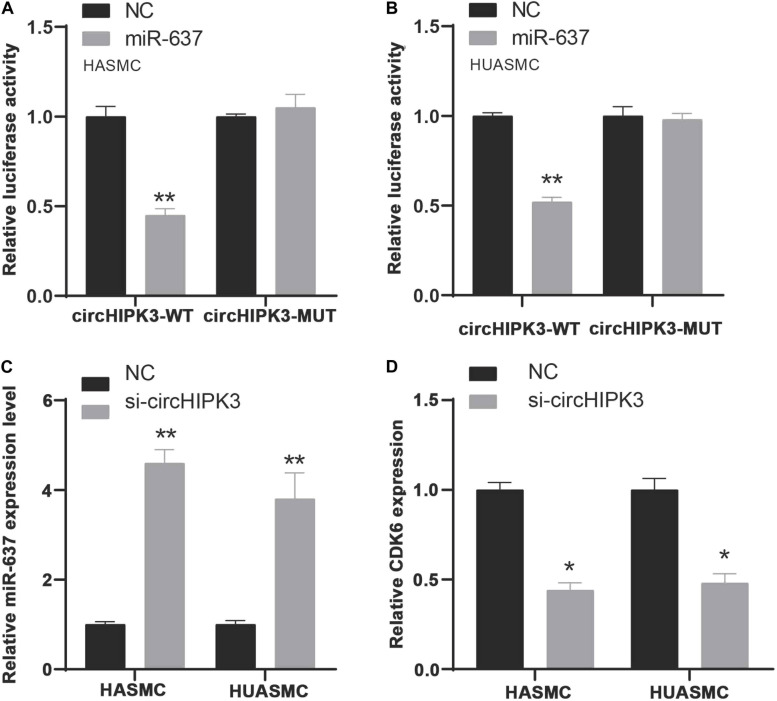
MiR-637/CDK6 was regulated by circHIPK3. **(A,B)** Luciferase assay detects miR-637 was targeted by circHIPK3 in HASMC and HUASMC cells. **(C)** RT-PCR analysis of miR-637 expression levels in HASMC and HUASMC after transfecting with si-circHIPK3. **(D)** RT-PCR analysis of CDK6 expression levels in HASMC and HUASMC after transfecting with si-circHIPK3. *P < 0.05, **P < 0.01.

To verify miR-637 was targeted by circHIPK3, we firstly applied a luciferase assay by co-transfecting miR-637 and luciferase reporters into VSMCs. The findings showed that the luciferase activity of circHIPK3-WT was significantly reduced compared to control group and circHIPK3-mutant group (Figures 7A,B). Moreover, it was observed that silencing of circHIPK3 up-regulated miR-637 expression (Figure 7C).

To confirm the effect of circHIPK3 on CDK6, CDK6 expression in HASMC and HUASMC cells transfected with sicircHIPK3 were detected using RT-PCR. CDK6 levels decreased after knockdown of circHIPK3 in VSMCs compared with the control group (Figure 7D). These data revealed that circHIPK3 and CDK6 interacted with miR-637 in VSMCs.

### Suppression of circHIPK3 Inhibited Cell Proliferation of VSMCs

**FIGURE 8:**
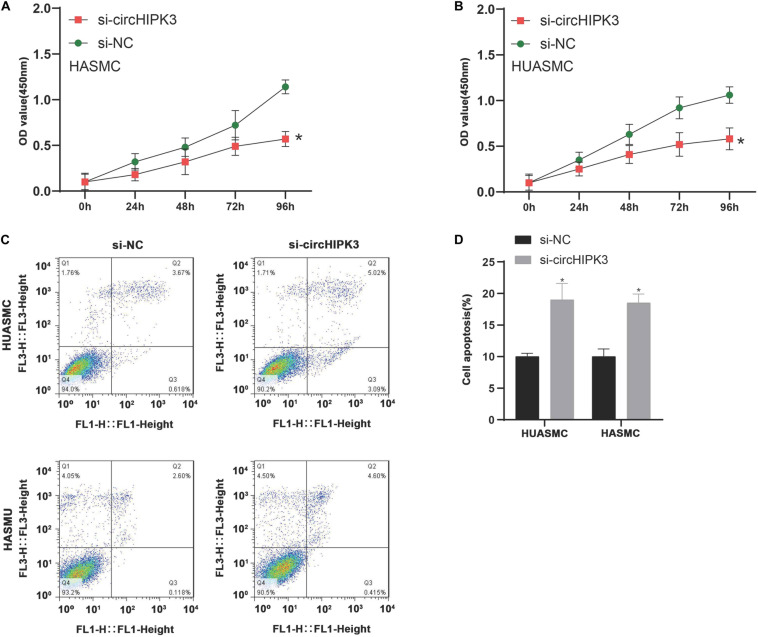
Suppression of circHIPK3 inhibited cell proliferation of VSMCs. **(A,B)** Suppression of circHIPK3 inhibited cell proliferation of VSMCs in HASMC and HUASMC cell. **(C,D)** Flow cytometry analysis of cell apoptosis in HASMC and HUASMC after transfecting with si-circHIPK3. *P < 0.05.

We used CCK-8 kit to detect the effect of circHIPK3 on cell proliferation, and the results indicated the cell proliferation rate in circHIPK3 knockdown cells were reduced compared to normal groups (Figures 8A,B). Based on flow cytometry analysis, it was found that knockdown of circHIPK3 considerably induced the apoptosis of HASMC and HUASMC cell when compared with the control group (Figures 8C,D). These findings suggested circHIPK3 could enhance cell proliferation ability and suppress cell apoptosis in VSMCs.

## Discussion

The mechanisms regulating atherosclerosis remain largely unclear. The present study aimed to identify differently expressed mRNAs in atherosclerosis by analyzing GSE6088 database. Our results revealed there were totally 467 increased DEGs and 490 decreased DEGs in atherosclerosis. Bioinformatics analysis showed The DEGs were mainly involved in modulating a series of pathways, including Glyoxylate and dicarboxylate metabolism, Tyrosine metabolism, Tryptophan metabolism, Beta-Alanine metabolism, Fatty acid biosynthesis, Starch and sucrose metabolism. Next, we constructed a PPI network to identify hub genes in atherosclerosis. Also, we identified CDK6 as a key regulator of atherosclerosis. The CDK6 kinase was correlated with cyclins D1, D2, and D3 and played a role in growth factor stimulation and cell cycle progression (Kozar and Sicinski, 2005). In this study, we found that CDK6 knockdown suppressed HASMC and HUASMC cell proliferation.

MiRNAs are an important class of cellular regulators in post-transcriptional gene regulation. However, miRNAs always interact with other regulatory factors to regulate cell metabolism by regulating downstream target genes, such as lncRNA and circRNA (Militello et al., 2016; Jin et al., 2018). Generally, overexpressed circRNAs inhibit the activity of miRNAs as sponge of miRNAs adsorbing it. Therefore, circRNA regulates the translation of downstream target genes by affecting the expression of miRNA, thus affecting the progress of the disease (Qu et al., 2015; Rong et al., 2017). To analyze the mechanism of circHIPK3 in VSMCs in modulating cell proliferation, cycle and apoptosis, we predicted and validated the target miRNA by bioinformatics and luciferase reporting experiments. It has been found that miR-637 was a target of circHIPK3 in VSMCs. It has been reported that miR-637 inhibited the human mesenchymal stem cells growth. MiR-637 was up-regulated during adipocyte differentiation and suppressed during osteoblast differentiation (Zhang et al., 2011). As a tumor suppressor gene, miR-637 plays a significant regulatory role in thyroid papillomatous carcinoma (Yuan et al., 2018), cervical cancer, glioma, cholangiocarcinoma, colorectal cancer and some other tumors (Li et al., 2018; Huang et al., 2019). In mechanism, we conducted luciferase reporting assay and found that the downstream target gene of miR-637 was CDK6.

Accumulating evidence suggests that circRNA is a broad and powerful regulator in cell progression. The role of circRNA in the atherosclerosis has gradually been recognized with the increasing number of sncRNA research. For instance, Holdt et al. (2016) reported that the presence of circANRIL could decrease atherosclerosis by inducing apoptosis, and suppress of proliferation. Shen et al. (2019) found that the expression of circRNA-0044073 was up-regulated and promoted the cell growth in atherosclerosis blood cells. CircRNAs play pivotal regulating roles for cell growth, cell cycle and apoptosis. For instance, circRNA BCRC4 has been found lower expression in bladder cancer tissues than in normal cases. However, over-expression of circBCRC4 induced apoptosis and suppressed BC viability via enhancing miR-101 and inhibiting the expression of EZH2 *in vitro* (Li et al., 2017). Moreover, it has been proved that hsa_circ_0001564 sponges miR-29c-3p to promote cell growth, stimulates cell cycle entry and inhibits cell apoptosis in osteosarcoma (Song and Li, 2018). Wang et al. confirmed a circRNA associated with the apoptosis of OSCC cells by constructing an apoptotic model and comparing with different expression profile. The experimental results showed that the silencing of circDOCK1 led to the increase of apoptosis by sponge miR-196a-5p in OSCC (Wang et al., 2018). In the present study, we knocked down circHIPK3 by transfecting si-circHIPK3 in VSMCs. The activity of VSMCs was detected by CCK-8, cell cycle and cell apoptosis assays. The study showed that down-regulation of circHIPK3 significantly influenced the progression of VSMCs, which implied that circHIPK3 played an important role in the tumorigenesis of VSMCs.

Atherosclerosis is the leading cause of death and disability in the developed countries. Although we are increasingly familiar with the disease, some fundamental characteristics and pathogenesis of the disease are still poorly understood. Our current study for the first time demonstrated a signaling axis circHIPK3/miR-637/CDK6 that might be responsible for the atherosclerotic progression. Specifically, circHIPK3 sponged miR-637 to reduce its expression in VSMCs and low-expression of miR-637 was supposed to lead to the increased CDK6 expression. And upregulated CDK6 was strongly involved in the enhanced proliferation of VSMCs, thereby accelerating the development of atherosclerotic. Our findings can contribute to understand the etiology of atherosclerosis well and provide a solid evidence base for future research.

Our research has certain limitations and needs to be improved in future study. First, we screed out DEGs according to GSE6088 database, the other significantly DEGs except CDK6 may also have important regulatory effects on atherosclerosis, and needs to be further studied. In addition, the depth of mechanism research is not sufficient, we only performed bioinformatic assay and *in vitro* functional experiments revealing the signaling axis circHIPK3/miR-637/CDK6 in atherosclerosis. Thus, in the follow-up research, we will further do *in vivo* assay to verify the mechanism of action of circHIPK3/miR-637/CDK6 axis.

Taken together, in this study, our results showed that CDK6 knockdown suppressed VSMCs cell proliferation. Further research found that circHIPK3s could act as a sponge of miR-637 and indirectly regulate the expression of CDK6 gene. The further study showed circHIPK3 could maintain cell cycle progression, reduce apoptosis and promote cell proliferation in smooth muscle cells. Therefore, circHIPK3 had a potential influence on atherosclerosis development. Our investigations revealed that CDK6 played a meaningful role in the progression of VSMCs, which could be regulated by the circHIPK3/miR-637 axis.

## Data Availability Statement

The datasets presented in this study can be found in online repositories. The names of the repository/repositories and accession number(s) can be found in the article/Supplementary Material.

## Author Contributions

YS and YZ: conception and design. LK, BH, and HJ: development of methodology. SL: sample collection. ZC and JS: analysis and interpretation of data. LK, BH, HJ, CW, and YS: writing, review, and/or revision of the manuscript. All authors contributed to the article and approved the submitted version.

## Conflict of Interest

The authors declare that the research was conducted in the absence of any commercial or financial relationships that could be construed as a potential conflict of interest.

## Footnotes

1 https://www.ncbi.nlm.nih.gov/geo/query/acc.cgi?acc=GSE6088

2 https://www.ncbi.nlm.nih.gov/geo/geo2r/

3 https://david.ncifcrf.gov/

4 http://string-db.org

5 www.targetscan.org
