# Supplementary figures and images for: Identification of Differently Expressed mRNAs in Atherosclerosis Reveals CDK6 Is Regulated by circHIPK3/miR-637 Axis and Promotes Cell Growth in Human Vascular Smooth Muscle Cells
Source: Front Genet. 2021 Feb 15;12:596169. doi: 10.3389/fgene.2021.596169 (PMC7917241; doi:10.3389/fgene.2021.596169)

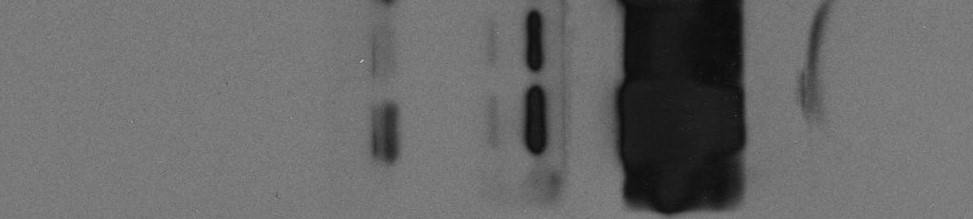

Supplement: Supplementary file 1 [file Image_1.JPEG]

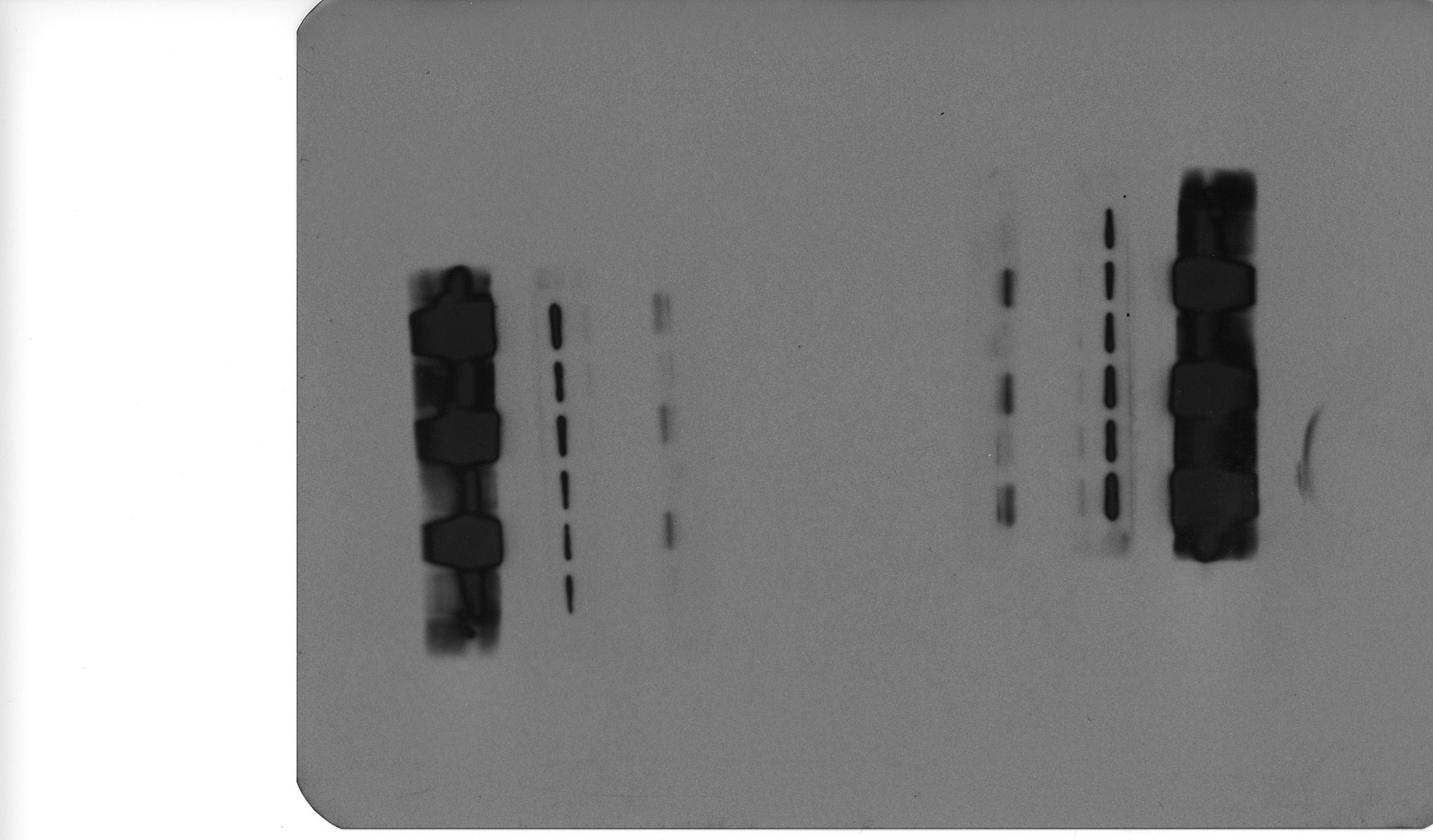

Supplement: Supplementary file 2 [file Image_2.JPEG]
